# Green preparation of anti-inflammation an injectable 3D porous hydrogel for speeding up deep second-degree scald wound healing†

**DOI:** 10.1039/d0ra04990e

**Published:** 2020-09-30

**Authors:** Xiao Xu, Lin Che, Lin Xu, Doudou Huang, Jiashen Wu, Zebang Du, Yuchun Lin, Xiaoqian Hu, Qingliang Zhao, Zhongning Lin, Ling Xu

**Affiliations:** State Key Laboratory of Molecular Vaccinology and Molecular Diagnostics, Center for Molecular Imaging and Translational Medicine, School of Public Health, Xiamen University Xiamen 361102 China lingxu@xmu.edu.cn linzhn@xmu.edu.cn zhaoql@xmu.edu.cn

## Abstract

Scalds are one of the most common injuries and the 4th cause of trauma globally. Alginate has emerged as a promising scald wound dressing. Herein, we present a facile applicable strategy for electron beam (EB) radiation crosslinking gelatin, alginate, and carboxymethyl cellulose (CMC) into an injectable three-dimensional (3D) porous hydrogel (3D-PH) with a double crosslinked network for reliable deep second-degree scald wound healing. In addition, the injectable 3D-PH stimulated proliferation and migration of dermal fibroblasts *in vitro* and the deep second-degree scald wound healing process is accelerated *in vivo*. Most importantly, *in vitro* results revealed that the injectable 3D-PH stimulated cell proliferation *via* inducing the expression of Ki-67, and suppressed inflammatory signals as indicated by the downregulation of inflammatory factors (IL-6, TNF-α) in L929 cells. We further demonstrated that the 3D-PGH accelerated the wound healing process of deep second-degree scald *in vivo*. This study indicated the injectable 3D-PH with a double crosslinked network could be applied as a multifunctional injectable scald wound dressing material for anti-inflammation, necrotic tissue-removal, and wound closure. These findings suggest that the injectable 3D-PH may be conducive to the evolution of new pharmaceuticals for burn wound healing.

## Abstract 2

The injectable 3D porous hydrogel with a double crosslinked network could be applied as a multifunctional injectable scald wound dressing material for anti-inflammation, necrotic tissue-removal, and wound closure.

## Graphical abstract

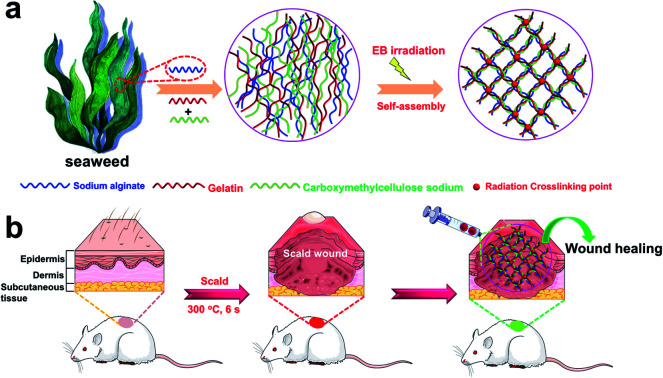

## 1. Introduction

Burns are a serious global public health problem. Annually, about 300 000 people die or suffer injuries caused by fires, scalds, electrical burns, and other forms of burns.^1^ Moreover, burns from scalds and fires account for approximately 80% of all reported burns.^2^ Scalds have some distinct pathophysiological characters among all types of burns, such as more wound exudate and necrotic tissue debris on the surface of the burn wound.^3^ The burn wound is classified into different degrees dependent upon the thickness of burn injuries.^4^ The lower layers of the dermis are severely damaged in deep second-degree burns.^5^ Deep second-degree scald wound healing is a complex physiological process in which skin tissue is repaired in the case of injury.^6^ During wound healing, the wound area is covered with necrotic tissue, and this inactive necrotic tissue could inhibit tissue repair. Also, high levels of inflammatory cytokines produced by necrotic tissue could affect cell migration and delay wound healing.^7,8^ It is urgent and critical to painlessly remove the necrotic and wound tissue debris. Therefore, hydrogel wound dressings are currently designed because of the similar structure to the natural extracellular matrix, which provides instructive environments for wound healing.^9–11^

In early 1960, O. Wichterle *et al.* reported the first case of hydrophilic gels,^12^ many studies have been focused on applications in wound healing *via* using hydrogels.^13–18^ Increased evidence showed hydrogels can deliver cytokines and growth factors,^19^ which is beneficial for burn wound healing. Also, hydrogels are good stain removers,^20–22^ and they are non-adherent and can engulf bacteria, which is benefits to three-dimensional (3D) network expansion upon exudate absorption.^23^ Hydrogels can meet many of the requirements for an ideal burn wound dressing, and sustain an ideal moist environment for healing while protecting the wound. It can be acted as a promising material for the treatment of burns and other skin lesions.^4,11^ However, conventional hydrogels are brittle, mechanically weak, and poorly deformable, which seriously hindered their further application in burn wound dressing.^24–26^ Dash *et al.* synthesized a gelatin hydrogel cross-linked by aldehyde-modified cellulose, in which the aldehyde groups could be reacted with amine groups of from gelatin through Schiff-base linkages to reinforce the network.^27^ However, the procedures to prepare the hydrogel are more laborious (24 h at room temperature and then 10 days at 4 °C). Thus, hydrogels developed by cheap materials and facile applicable method with burn wound healing ability are highly anticipated, while it remains a challenge.

Gelatin, a readily available and relatively inexpensive material, which has been widely used as hydrogel scaffolds for tissue engineering due to their good cell attachment properties.^9^ Alginate is also a prominent material for wound dressings as well, especially for the treatment of the deep second-degree scald wound, which mainly attributes to exudate absorption potential and ability to maintain a moist wound environment.^28^ In addition, alginates have favorable biological properties, such as biocompatibility, non-antigenicity, and biodegradability.^29,30^ Besides, carboxymethyl cellulose (CMC) with a large number of carboxymethyl groups, which has been widely used as a natural ingredient for hydrogels mostly because of these advantages.^31,32^ Gelatin and alginate can induce platelet activation for wound hemostasis, and CMC can reduce the amount of exudate uptake during skin epithelialization. In this work, gelatin, alginate, and CMC were chosen because combining their advantages compensates for the lack of application of one or both of these natural polymers.

**Fig. 1:**
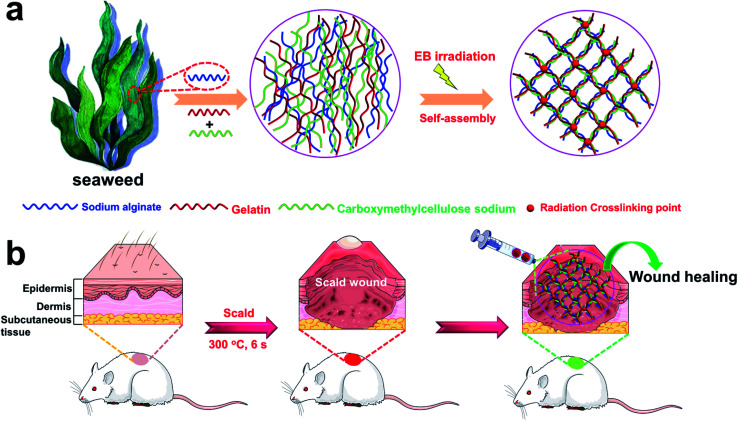
Schematic illustrations of the crosslinking process for the production of 3D-PH. (a) Schematic diagram of the preparation of 3D-PH. (b) Schematic of the injectable 3D-PH *in vivo* application of for deep second-degree scald wound healing.

Radiation crosslinking is considered an ideal technique for hydrogel preparation without cytotoxic additives.^33^ In contrast to conventional chemical cross-linking, neither initiators nor cross-linkers are required when using radiation crosslinking, which results in a product free of toxic additives. Thus, irradiation sterilization is the best method for the terminal sterilization of medical products approved by the Food and Drug Administration (FDA). In view of this, herein, the introduction of electron beam (EB) radiation crosslinking technology to both alginate, gelatin, and CMC allows the formation of an injectable 3D porous hydrogel (3D-PH) with double crosslinked network upon applying EB-irradiation, which is a straightforward, fast, and cost-effective approach (Fig. 1a). This study focused on the synthesis and comprehensive characterization of environmentally friendly hydrogels based on gelatin, CMC, and alginate for burn wound repair substitutes. The results from the Fourier transform infrared (FT-IR) and scanning electron microscopy (SEM) analyses indicated that the injectable 3D-PH with Schiff base group was successfully prepared. The thermogravimetric analysis (TGA) showed that the gelatin–alginate hydrogel had good thermal stability. *In vitro* results demonstrated that the injectable 3D-PH stimulates cell proliferation and suppresses the activation of inflammatory signals of L929 cells, which means that injectable 3D-PH has good biocompatibility. Furthermore, compared to a commercially available Coloplast wound dressing, the wound healing rates of the gelatin–alginate hydrogel were significantly higher than that of the Coloplast wound dressing group. These results suggested that the injectable 3D-PH could be used to accelerated deep second-degree scald wound healing.

## 2. Experimental

### 2.1 Materials

Gelatin (*M*_n_ = 50 000–100 000) from porcine skin, type A, gel strength ∼300 g bloom, was obtained from Sigma Chemical Co. Ltd. Sodium alginate (pharmaceutical grade) was purchased from Qingdao Bright Moon Seaweed Group Co. Ltd., China. Carboxymethylcellulose sodium (pharmaceutical grade) (CMC) was purchased from Xi'an Yue Lai Medical Technology Co. Ltd., China. Commercially available Coloplast wound dressing (Coloplast Co. Ltd., China) was used in the deep second-degree scald wound healing as control. All deep second-degree scald wounds were covered with commercially available transparent film dressing (Tegaderm Film, 3M, MN) to prevent loss of moisture in the hydrogel. All other reagents were used without further purification. Deionized water (PALL, Cascada BIO) was used for all experiments unless otherwise stated.

### 2.2 Preparation of the 3D-PH

The preparation scheme for the 3D-PH is outlined in detail in Fig. 1a. The gelatin–alginate–CMC solution (6 wt%) were typically prepared by adding gelatin, sodium alginate, and carboxymethylcellulose sodium powders (different weight ratio of 2 : 1 : 3 (GSC213), 2 : 2 : 2 (GSC222) and 3 : 2 : 1 (GSC231)) to 50 mL of deionized water and stirring in a water bath for 15 min at 50 °C until complete solubilization. The gelatin–alginate–CMC solution was separately packed into a test tube for centrifugation to remove bubbles. Subsequently, inject the gelatin–alginate–CMC solution into the syringe (10 mL). The 3D-PH was prepared *via* EB radiation crosslinking gelatin–alginate–CMC solution at room temperature over the range of 10 kGy.

### 2.3 Characteristics of 3D-PH

3D-PHs were freeze-dried and then characterized accordingly. Fourier-transform infrared (FT-IR) spectra were collected on a Nicolet Avatar 370 FTIR spectrometer (Thermo Nicolet Company, USA) in attenuated total reflectance mode with a resolution of 4 cm^−1^ and 32 scans. The thermogravimetric analysis (TGA) was performed (NETZSCH, TG209, F3) in the temperature range from 25 to 800 °C with a heating rate of 10 °C per minute under a nitrogen flow. The surface and cross-sectional morphologies of the 3D-PHs were performed using field-emission scanning electron microscopy (SEM) (JSM-6390 LV, JEOL, Japan). All the 3D-PHs were sputtered with gold to enhance the electron conductivity before observation by SEM. The compressive strength of samples was tested by the texture analyzer. The cup (the capacity of 100 mL) was firmly fixed on the sample stage. And samples were not moved from the cup and it was ensured that the height of the samples were identical by cut at least 3 cm away from debridement glue surface. The flat plate probe (P/0.52 Delrin cylinder probe) with 5 cm of diameter was attached to the sample surface and moved downward vertically. The testing was performed with the pretest speed of 3.0 mm s^−1^, the test speed of 1.0 mm s^−1^, the post speed of 3.0 mm s^−1^, and the drop height of 3 cm.

### 2.4 Cell culture

The mouse fibroblast cell line L929 was obtained from ATCC (Manassas, VA, USA) and cultured in Dulbecco's modified eagle's medium (DMEM) (Hyclone, UT, USA) supplemented with 10% fetal bovine serum (FBS) and 1% penicillin–streptomycin. Cells were cultured in a humidified incubator with 5% CO_2_ at 37 °C.

### 2.5 Cell viability assay

Cells were plated in 96-well plates (8 × 103 cells per well) and incubated for 24 h. After treatment with various concentrations of GSC222 (0.06125–0.5 mg L^−1^) for 24 h. 20 μL of MTS (Madison, WI, USA) was added to per well and incubated for 2 h. The optical density (OD) of each well was measured spectrophotometrically at 490 nm by a multifunctional microplate reader (BMG LRBTECH CLARIOstar, Offenburg, Germany).

### 2.6 Western blot analysis

Extractions of proteins from L929 cells and western blotting were performed as described previously.^34^ The following primary antibodies were used: anti-Ki67 (Beyotime, 1 : 000), anti-IL-6, and anti-TNF-α (CST, 1 : 1000), and anti-GAPDH (Kangchen, 1 : 10 000). The membranes were probed with secondary antibody for an hour and were visualized using the Azure Biosystems (Beijing, China) with enhanced chemiluminescence Kit (Thermo).

### 2.7 Immunofluorescence assay

Immunofluorescence analysis was performed as described previously.^35^ Briefly, the cells were fixed with 4% (w/v) paraformaldehyde (pH 7.4) for 30 min followed by permeabilization with 0.3% Triton™ X-100 in PBS for 5 min. Then, the cells were blocked with 1% bovine serum albumin (BSA) for 45 min at room temperature. After incubating with the primary antibodies (anti-Ki67, anti-IL-6, or anti-TNF-α) at 4 °C overnight, the samples were incubated with goat anti-mouse/rabbit IgG fluorescent secondary antibody for 1 h and counterstained with DAPI. Finally, stained cells were examined using a confocal laser scanning microscope (Zeiss LSM 780, Carl Zeiss, Jena, Germany) equipped with ×63 oil objective.

### 2.8 Deep second-degree scald wound healing model

All studies adhered to procedures consistent with the International Guiding Principles for Biomedical Research Involving Animals issued by the Council for the International Organizations of Medical Sciences (CIOMS). All animal procedures were performed in accordance with the Guidelines for Care and Use of Laboratory Animals of Xiamen University and approved by the Animal Ethics Committee of Xiamen University. The male Sprague-Dawley (SD) rats (250 ± 20 g) were obtained from Shanghai SLAC Laboratory Animal Co., Ltd., and all animals were cared for and then treated according to the instructions and approval of the Institutional Animal Care and Use Committee of Xiamen University. All animals were housed in standard cages with food and water available *ad libitum* in a specific pathogen-free facility. Animals were allowed to acclimatize for one week before experimentation.

The SD rats model with deep second-degree scald was constructed. Briefly, SD rats were first anesthetized with an intraperitoneal injection of chloral hydrate, the back hair of the rat was shaved and the skin was disinfected using ethanol. Then, a preheated bar with a temperature of 300 °C was applied to the back of each rat for 6 s to create three 8 mm diameter burns on both sides of the spine. Optical coherence tomography (OCT) images showed that the cross-sectional skin tissue of rats was obviously damaged after scald, and the model of deep second-degree scald was successfully established (Fig. S2†). For the GSC 222 3D-PH treated wounds, 1 mL 3D-PH was injected into the scald wounds by 10 mL syringes. All scald wounds were covered with commercially available transparent film dressing (Tegaderm Film, 3M, MN) to prevent loss of moisture. Photographs were taken after dressing change and the scald wound area was measured using ImageJ software (Bethesda, MA, USA). The percentage of the original scald wound area at different time points was calculated by comparing them to the wound area on the day of surgery.

### 2.9 Spectral-domain optical coherence tomography (SD-OCT) imaging the skin *in vivo*

All the experiments were conducted on a home-built OCT system.^36^ The skin of male SD rats was placed in a photosensitive polymer 3D-printed groove module to reduce the effect of micromotion. It was then fixed on an X–Y–Z adjusting platform for the easy adjustment during imaging. The sample arm was adjusted to allow the beam to focus on the scald wound. The position of the SD rats was adjusted to ensure that the signal-to-noise ratio (SNR) and contrast of the 2D images were optimal.

### 2.10 Histological analysis

Twenty days after the surgery, the skin tissue of rats was fixed with 4% (w/v) paraformaldehyde solution (pH 7.4) for 48 h, and dehydrated in a graded series of ethanol (70%, 80%, 90%, and 100%) and then embedded in paraffin, which used for sliced into a section with 5 μm of thickness by a microtome. Subsequently, stained with hematoxylin–eosin (H&E). The stained tissue was observed and recorded by an optical inverted microscope (TS-100, Nikon, Tokyo, Japan).

### 2.11 Immunohistofluorescence analysis

Skin tissue sections were incubated in 1% Triton X-100/PBS solution for 30 min and blocked with 1% bovine serum albumin (BSA) for 1 hour at room temperature, followed by incubation with the primary antibodies (anti-Ki67, anti-IL-6, or anti-TNF-α) at 4 °C overnight. The samples were incubated with a goat anti-mouse/rabbit IgG fluorescent secondary antibody for 1 h and nuclei were stained with DAPI. The sections were visualized by a confocal laser scanning microscope (Zeiss LSM 780, Carl Zeiss, Jena, Germany).

### 2.12 Statistical analysis

All experimental data in the experiment were expressed as mean ± standard deviation. Data analyses were performed using the SPSS 17.0 software (SPSS, IL, USA). Statistical analyses were assessed by using a student's *t*-test and one-way analysis of variance (ANOVA). The differences were considered statistically significant at the value of *P* < 0.05.

## 3. Results and discussion

In briefly, the gelatin–alginate–CMC solution (6 wt%) were typically prepared by adding gelatin, sodium alginate, and carboxymethylcellulose sodium powders (different weight ratio of 2 : 1 : 3 (GSC213), 2 : 2 : 2 (GSC222) and 3 : 2 : 1 (GSC231)) to 50 mL of deionized water and stirring in a water bath for 15 min at 50 °C until complete solubilization. Subsequently, inject the gelatin–alginate–CMC solution into the syringe (10 mL). Three different proportions of 3D-PH were prepared *via* EB radiation crosslinking gelatin–alginate–CMC solution for 5 min at room temperature over the range of 10 kGy (Fig. 1a).

**Fig. 2:**
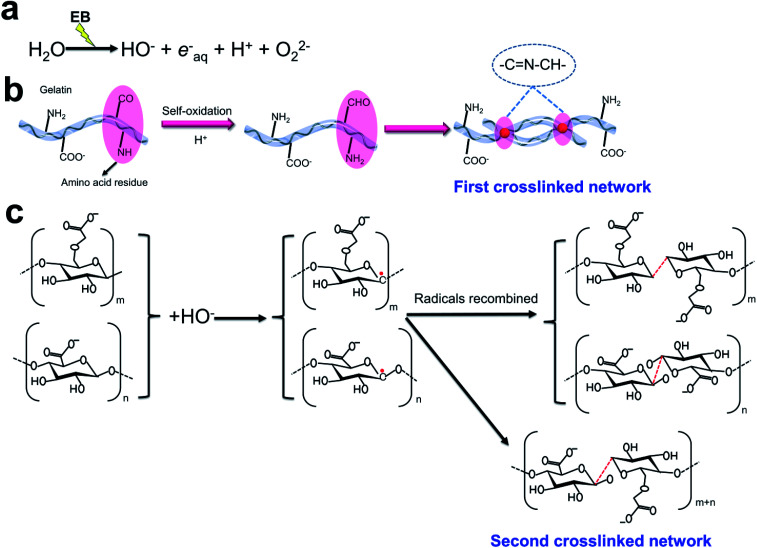
Mechanism of EB radiation crosslinking of injectable 3D-PH. (a) Ionizing radiation reaction equation of water in polymer aqueous solution. (b) Gelatin self-crosslinking to form the first network of injectable 3D-PH. (c) The crosslinking mechanism of alginate and CMC under EB-irradiation.

The EB radiation crosslinking mechanism in Fig. 2 was proposed for the crosslinking of injectable 3D-PH. The radiation energy of EB is mostly absorbed by water in aqueous solutions, and the radiolysis of water mainly yields reactive species such as hydroxyl radical (OH^−^), proton radical (H^+^), hydrated electron (e_aq_^−^), and superoxide (O_2_^−^) (Fig. 2a).^37^ The amino acid residues in the gelatin molecule are prone to self-oxidation to form an aldehyde group, which can be crosslinked with the amino acid on the gelatin molecule to form Schiff base group (Fig. 2b), this is the first crosslinked network in the injectable 3D-PH. Furthermore, the OH^−^ is conceived as a very reactive species, which can be removed the H in the carbon chains of alginate and CMC, inducing the formation of alginate-derived radicals, CMC-derived radicals, and H_2_O. Subsequently, the radicals recombined to form a new covalent bond between the carbon chains (Fig. 2c), which is the second crosslinked network. Hydrogen bonds formed between injectable 3D-PH stabilize the chemical structure of hydrogels. The new bonds formed during electron beam irradiation make the molecular chains of hydrogels connect more closely. The double crosslinked network structure of EB-triggered and Schiff bases can significantly strengthen the hydrogel. These results indicated that a stable double crosslinked network structure can be formed by EB irradiation crosslinking injectable 3D-PH.

**Fig. 3:**
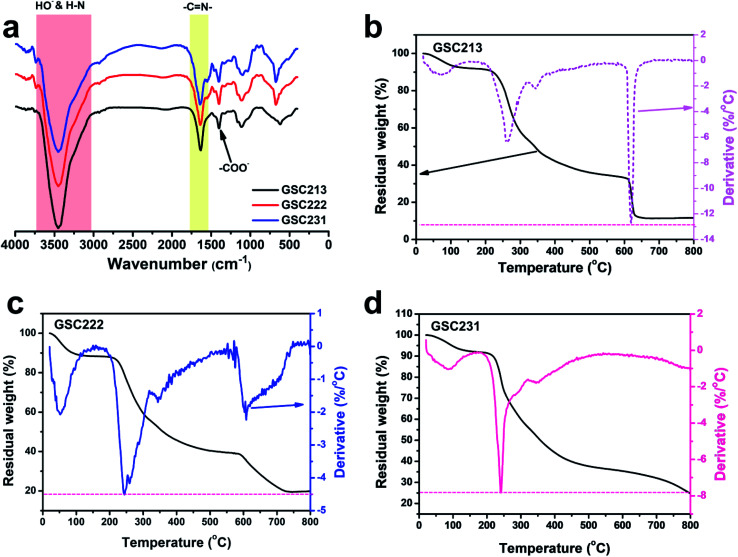
Characterization of injectable 3D-PH. (a) FT-IR spectra of GSC213, GSC222, GSC231, and GSC222 (10 kGy) injectable 3D-PH. TGA and DTG curves for (b) GSC213, (c) GSC222, and (d) GSC231 injectable 3D-PH.

The chemical structure changes that appeared after EB irradiation in the injectable 3D-PH was investigated by FT-IR spectroscopy (Fig. 3a). The spectra of GSC222, GSC213, GSC231 injectable 3D-PH (10 kGy) showed a characteristic absorption band located at 3448.5 cm^−1^ assigned to the HO– and H–N groups. The characteristic absorption bands at 1640.1 and 1400.6 cm^−1^ were attributed to the adsorption of –C=N– (Schiff base group) and –COO–, respectively. The FT-IR results indicated that EB irradiation did not destroy the Schiff base group in the injectable 3D-PH after crosslinking.

The TGA and derivative thermogravimetry (DTG) curves for the GSC213, GSC222, and GSC231 injectable 3D-PH presented in Fig. 3b–d show that the obtained injectable 3D-PH have good thermal stabilities. The TGA-DTG curve showed that the first region, between 25 and 100 °C, corresponded to the evaporation of physisorbed water, which was due to the 3D-PH with good hydrophilicity. The TGA-DTG curves (Fig. 3b) of the GSC213 gelatin–alginate hydrogel revealed three main decomposition processes. The first occurred in the temperature range from 195 to 315 °C, while the second one occurred from 315 to 367 °C, and the third one occurred from 580 to 660 °C. The weight loss was due to the complex thermal decomposition of the carboxylic groups, –C=N–, and C–O–C. For the GSC222 gelatin–alginate hydrogel (Fig. 3c), four regions of weight loss, which was similar to GSC213 gelatin–alginate hydrogel. The GSC231 gelatin–alginate hydrogel became less thermally stable compared to the GSC222 gelatin–alginate hydrogel. The peaks in the degradation range from 580 to 660 °C almost disappear due to the decrease in CMC (Fig. 3d). The chemical and thermal stabilities of the molecule chain suggest that the injectable 3D-PH is suitable for biomedical materials.

**Fig. 4:**
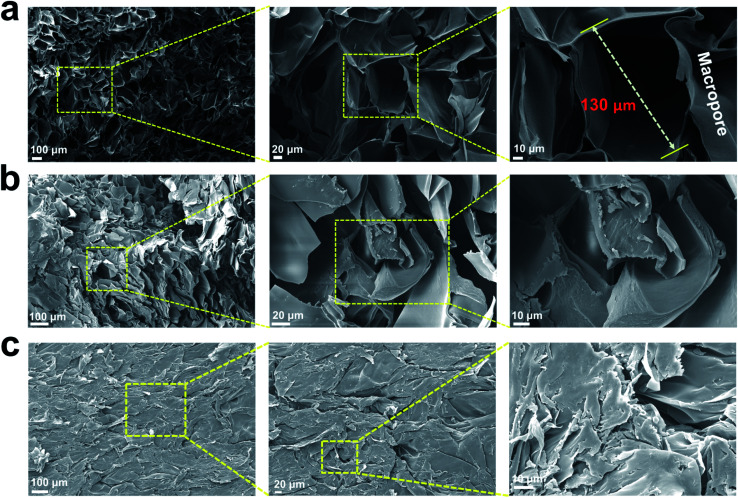
Surface morphology characterization for injectable 3D-PH. SEM images of the (a) GSC222, (b) GSC213, and (c) GSC231 injectable 3D-PH at different magnifications.

SEM images of the prepared injectable 3D-PH (Fig. 4) displayed their surface morphology. The GSC222 gelatin–alginate hydrogel formed was 3D porous, and lots of macropores cross-linked with each other forming a 3D porous network structure, and the pores were distributed uniformly and their diameter was about 130 μm. Maintaining 3D porous structures in hydrogels is important in wound healing, as dense, the porous material can absorb wound exudates and largely block the escape of red blood cells and platelets, while maintaining a suitably moist environment for effective wound healing.^38,39^ Furthermore, the 3D porous network structure facilitates cell adhesion, growth, and proliferation, which is good at accelerated wound healing. The SEM-observed pore size of GSC222 gelatin–alginate hydrogel was microscale, and denser than GSC213 and GSC231 injectable 3D-PH.

As shown in Fig. S1,† before EB radiation, when the gel sample is subjected to force, the force curve changes smoothly, compression, and a tensile curve are symmetric. After irradiation, the gel sample is subjected to a tortuous force curve, and the viscosity curve significantly reduced, indicating that the sample presents a spatial structure after irradiation crosslinking, with brittleness, after the force, the spatial structure is destroyed, the uneven force resulting in fluctuation of the force curve. This is conducive to the maintenance of the tissue structure, the formation of skin tissue scaffolding, and the promotion of wound healing when the debridement dressing is applied to the skin.

**Fig. 5:**
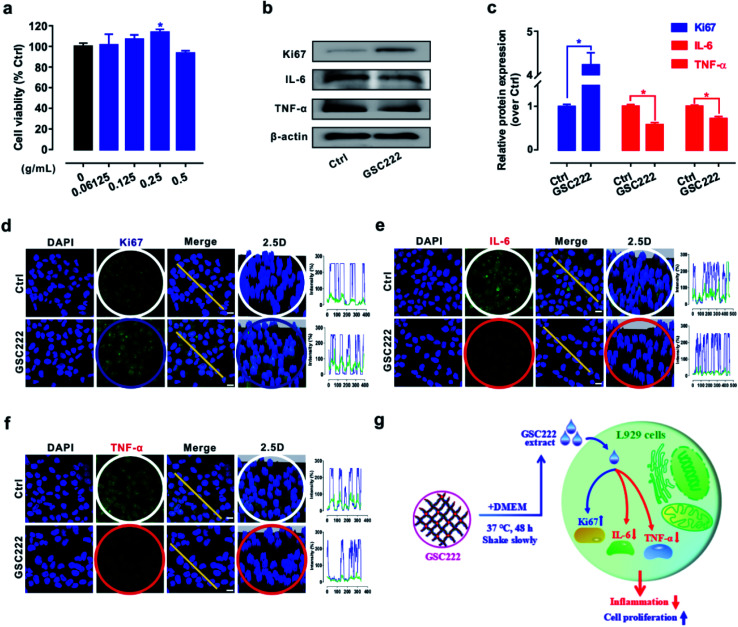
3D-PH stimulate cell proliferation and suppresses inflammation in L929 cells. (a) Cell viability was determined using a MTS assay in L929 cells treated with GSC222 injectable 3D-PH at different concentrations (0, 0.06125, 0.125, 0.25, and 0.5 g mL^−1^) for 24 h. (b and c) Representative immunoblot and quantification analysis of Ki67, IL-6, and TNF-α in L929 cells treated with GSC222 injectable 3D-PH (0.25 g mL^−1^) for 24 h. β-Actin was used as an internal standard for protein loading. (d–f) Representative images of Ki67 (d), IL-6 (e) and TNF-α (f) puncta (green) and DAPI (blue) in L929 cells treated with GSC222 injectable 3D-PH (0.25 g mL^−1^) for 24 h. Scale bar: 10 μm. (g) An illustration for GSC222 injectable 3D-PH mediated cell proliferation and interference of inflammation signal activation in L929 cells. Data were expressed as mean ± SD. *P < 0.05, *versus* the control group.

Hydrogel wound dressings can provide a moist local environment for the wound surface, promoting skin renewal and cell proliferation making them increasingly one of the best treatment options to promote wound healing.^40,41^ However, excessive activation of inflammatory signals can cause chronic inflammation and thus impair cutaneous wound healing.^42^ Therefore, promote cell proliferation and block inflammation signal activation using wound dressing materials may offer powerful new treatment modalities for wound healing. So it was of interest to investigate whether or not the 3D-PH plays a critical role in promoting cell proliferation and blocking inflammation signal activation *in vitro*. As shown in Fig. 5a, the cell viability was significantly increased by treatments with 3D-PH (GSC222) at 0.25 g mL^−1^ for 24 h. Western blot results show that the levels of cell proliferation-related protein Ki-67 were elevated, while the inflammatory factors (IL-6, TNF-α) were reduced (Fig. 5b and c) in GSC222 treated L929 cells. Cell imaging analysis to quantify the protein distribution and content in cells is a well-accepted method to study the function of the protein.^43^ Further, to locate the different states of Ki-67, IL-6, and TNF-α in L929 cells. Firstly, we stained nuclei with DAPI green to locate the content of Ki-67, IL-6, and TNF-α. As shown in Fig. 5d–f of the confocal laser scanning microscopy images, the results showed that the fluorescence puncta of Ki67 were up-regulated (Fig. 5d), while the fluorescence puncta of IL-6 and TNF-α were down-regulated in GSC222 treated L929 cells (Fig. 5d and e). In general, these results demonstrated that the 3D-PH stimulates cell proliferation and suppresses the activation of inflammatory signals of L929 cells (Fig. 5g).

**Fig. 6:**
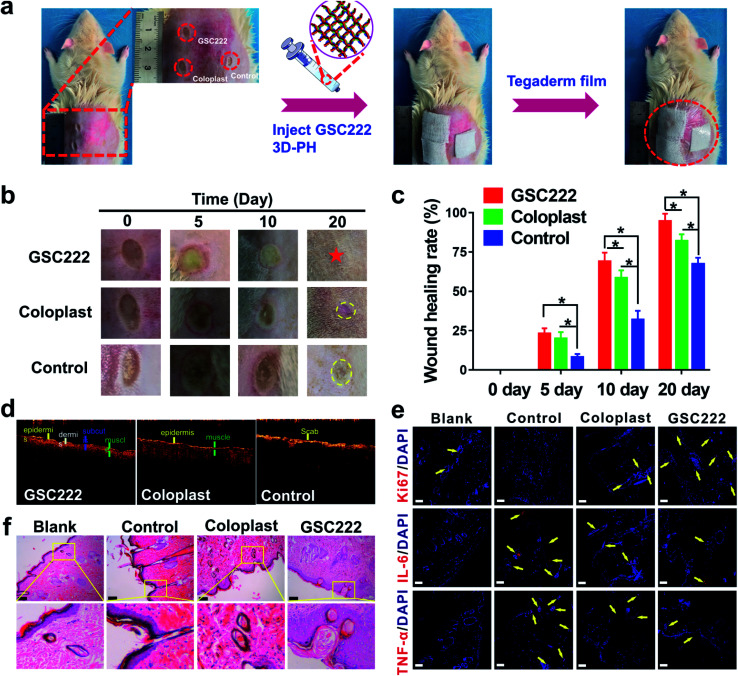
Wound closure of 3D-PH treated deep second-degree scald wound healing in the rat. (a) Schematic diagram of the surgical procedure. (b) *In vivo* study depicting GSC222 injectable 3D-PH, Coloplast wound dressing, and control photograph of rat deep second-degree scald wounds at days 0, 5, 10, and 20. (c) Statistics of the wound-healing rates for various treatments. (d) OCT images depicting GSC222 injectable 3D-PH, Coloplast wound dressing, and control photograph of rat deep second-degree scald wounds on days 20. (e) Ki67, IL-6, and TNF-α immunofluorescence staining of histological sections. The yellow arrow represents the protein expression position. DAPI, 4A, 6-diamidino-2-phenylindole. (f) Histological assessment of the deep second-degree scald wounds at days 20 *via* H&E stained. The lengths of the scale bars, 50 μm.

To investigate that the GSC222 injectable 3D-PH having the capability to accelerate necrotic tissue removal and wound healing *in vivo*. As shown in Fig. 1b, the GSC222 injectable 3D-PH *in vivo* application of for deep second-degree scald wound healing. The deep second-degree scald model was created on the backs of the rats by a heated flat plate probe at 300 °C and 6 s. As shown in Fig. 6a, three wounds were constructed on the backs of the rats, the GSC222 injectable 3D-PH, the Coloplast wound dressing, and the blank control were injected into three groups of scald wounds. In addition, Tegaderm film was employed to prevent loss of moisture. As can be observed from the photo of the wound healing process (Fig. 6b). After 10 d of treatment, compared with blank control, the wounds treated by the GSC222 injectable 3D-PH, the Coloplast wound dressing was dry and the scabs have formed (Fig. 6b). The healing rates of the GSC222 injectable 3D-PH, the Coloplast wound dressing were significantly higher than that of the blank control group (Fig. 6c). After 20 d of treatment, the defections of the wounds treated by the GSC222 injectable 3D-PH was almost invisible, and the healing rates of the GSC222 injectable 3D-PH were significantly higher than that of the Coloplast wound dressing group (Fig. 6b and c). The OCT images of the cross-sectional skin of the rats showed that the wound healing effect of GSC222 3D-PH treatment was significantly better than that of the Coloplast wound dressing group and control group (Fig. 6d). We next examined histopathological changes in the skin to determine whether the GSC222 injectable 3D-PH could mitigate deep second-degree scald-induced morphological damage in the skin. As shown in Fig. 6f, the GSC222 injectable 3D-PH and the Coloplast wound dressing treatment induced marked tissue repair and angiogenesis in the skin, and the GSC222 injectable 3D-PH treatment is more significant. Furthermore, immunohistofluorescence analysis showed that the fluorescence puncta of Ki67 were up-regulated, while the fluorescence puncta of IL-6 and TNF-α were down-regulated in the GSC222 injectable 3D-PH and the Coloplast wound dressing treatment groups (Fig. 6e). In general, these results demonstrated that the GSC222 injectable 3D-PH may be accelerated wound healing process *via* suppressing the activation of inflammatory signals *in vivo*.

## 4. Conclusions

In summary, a novel injectable 3D-PH with a double crosslinked network *via* EB radiation crosslinking was developed to accelerate the deep second-degree scald wound healing and inhibit infection. The double crosslinked network structure and Schiff bases can significantly strengthen the injectable 3D-PH hydrogel. Importantly, the injectable 3D-PH with a double crosslinked network could be injected and completely cover the irregular-shaped wounds to absorb wound exudates and largely block the escape of red blood cells and platelets, while maintaining a suitably moist environment for effective wound healing. The injectable 3D-PH plays a critical role in promoting cell proliferation and blocking inflammation signal activation *in vitro* and *in vivo*. Compared with commercial Coloplast wound dressing, the scald wound healing better after GSC222 3D-PH treatment. This work presents a novel injectable 3D-PH with multiple advantages and good potential as a wound dressing material for deep second-degree scald patients.

## Conflicts of interest

There are no conflicts to declare.

## Supplementary Material

RA-010-D0RA04990E-s001
