# Supplementary material for: Green preparation of anti-inflammation an injectable 3D porous hydrogel for speeding up deep second-degree scald wound healing
Source: RSC Adv. 2020 Sep 30;10(59):36101–10. doi: 10.1039/d0ra04990e (PMC9056965; doi:10.1039/d0ra04990e)
Supplement: RA-010-D0RA04990E-s001 [file RA-010-D0RA04990E-s001.pdf]

Electronic Supplementary Material (ESI) for RSC Advances.
This journal is © The Royal Society of Chemistry 2020

Electronic supplementary information

Green preparation of anti-inflammation injectable 3D porous hydrogel for speed up deep second-degree scald wound healing

Xiao Xu[†], *Lin Che*[†], *Lin Xu, Doudou Huang, Jiashen Wu, Zebang Du, Yuchun Lin, Xiaoqian Hu, Qingliang Zhao*[*], *Zhongning Lin*[*], *and Ling Xu*[*]

State Key Laboratory of Molecular Vaccinology and Molecular Diagnostics & Center for Molecular Imaging and Translational Medicine, School of Public Health, Xiamen University, Xiamen 361102, China

[†]These authors contributed equally to this work.

[*]Corresponding author.

E-mail: zhaoql@xmu.edu.cn, linzhn@xmu.edu.cn, and lingxu@xmu.edu.cn.

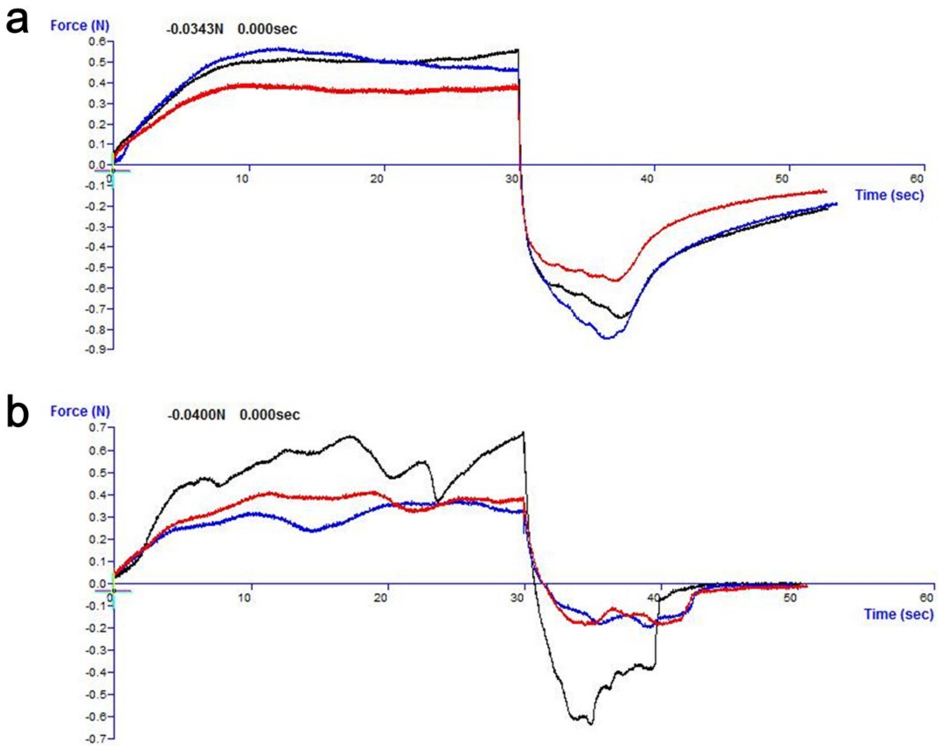

Figure S1. Figure S1. The compressive strength of GSC213 (BLACK), GSC222 (BLUE), and GSC231 (RED) for (a) before and (b) after EB radiation.

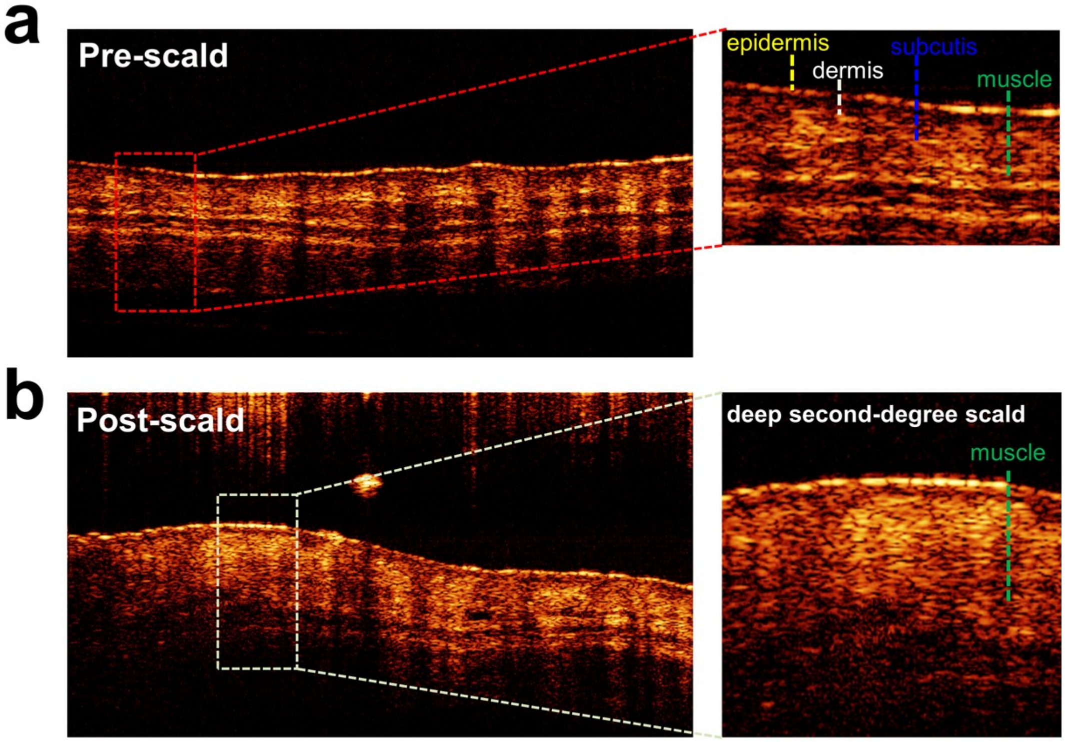

Figure S2. OCT images of the (a) pre-scald and (b) post-scald wound.

References

1. L. Che, H. Yao, C.-L. Yang, N.-J. Guo, J. Huang, Z.-L. Wu, L.-Y. Zhang, Y.-Y. Chen, G. Liu, Z.-N. Lin and Y.-C. Lin, *Nanotoxicology*, 2020, **14**, 162-180.
2. S. Zhang, L. Che, C. He, J. Huang, N. Guo, J. Shi, Y. Lin and Z. Lin, Cell Death Dis., 2019, 10, 523.
3. Y. Huang, M. Li, D. Huang, Q. Qiu, W. Lin, J. Liu, W. Yang, Y. Yao, G. Yan, N. Qu, V. V. Tuchin, S. Fan, G. Liu, Q. Zhao and X. Chen, *Small*, 2019, **15**, e1902346.
